# Jellyfish Bioprospecting in the Mediterranean Sea: Antioxidant and Lysozyme-Like Activities from *Aurelia coerulea* (Cnidaria, Scyphozoa) Extracts

**DOI:** 10.3390/md19110619

**Published:** 2021-10-31

**Authors:** Loredana Stabili, Lucia Rizzo, Rosa Caprioli, Antonella Leone, Stefano Piraino

**Affiliations:** 1 Department of Biological and Environmental Sciences and Technologies, University of Salento, Via Prov.le Lecce Monteroni, 73100 Lecce, Italy; rosa.caprioli@unisalento.it (R.C.); stefano.piraino@unisalento.it (S.P.); 2 Institute of Water Research, National Research Council, S.S. di Taranto, Via Roma 3, 74123 Taranto, Italy; 3 Department of Integrative Marine Ecology, Stazione Zoologica Anton Dohrn, Villa Comunale, 80121 Napoli, Italy; 4 Institute of Sciences of Food Production, National Research Council (CNR-ISPA), Via Prov.le Lecce Monteroni, 72100 Lecce, Italy; antonella.leone@ispa.cnr.it; 5 Consorzio Nazionale Interuniversitario per le Scienze del Mare (CoNISMa), Piazzale Flaminio 9, 00196 Roma, Italy

**Keywords:** bioactive compounds, antimicrobial compounds, lysozyme-like activity, peptides, moon medusa

## Abstract

Marine invertebrates represent a vast, untapped source of bioactive compounds. Cnidarians are represented by nearly 10,000 species that contain a complex mixture of venoms, collagen, and other bioactive compounds, including enzymes, oligosaccharides, fatty acids, and lipophilic molecules. Due to their high abundance in coastal waters, several jellyfish taxa may be regarded as candidate targets for the discovery of novel lead molecules and biomaterials and as a potential source of food/feed ingredients. The moon jellyfish *Aurelia coerulea* is one of the most common jellyfish worldwide and is particularly abundant in sheltered coastal lagoons and marinas of the Mediterranean Sea, where it first appeared—as an alien species—in the last century, when Pacific oyster cultivation began. In the present study, the antioxidant and lysozyme antibacterial activities associated with extracts from different medusa compartments—namely the umbrella, oral arms, and secreted mucus—were investigated. Extracts from the oral arms of *A. coerulea* displayed significant antioxidant activity. Similarly, lysozyme-like activity was the highest in extracts from oral arms. These findings suggest that *A. coerulea* outbreaks may be used in the search for novel cytolytic and cytotoxic products against marine bacteria. The geographically wide occurrence and the seasonally high abundance of *A. coerulea* populations in coastal waters envisage and stimulate the search for biotechnological applications of jellyfish biomasses in the pharmaceutical, nutritional, and nutraceutical sectors.

## 1. Introduction

Marine invertebrates represent a source of bioactive compounds that are generally used as defensive barriers against predators, parasites, and microbial pathogens or as messengers for intraspecific and interspecific communication. The enormous chemical diversity of marine invertebrate products guarantees an almost unlimited resource in the search for novel bioactive molecules [1] for pharmacological applications. Cnidarians are a large taxonomic group that is constituted by nearly 10,000 marine species that are characterized by highly specialized mechano-sensory cells (cnidocytes) containing proteinaceous venomous mixtures that are used both for both prey capture and defence against predators. Lacking adaptive immunity, cnidarians have an array of first-line defence mechanisms that are addressed to the recognition and neutralization of invaders [2]. Several toxic compounds isolated from cnidarian body extracts have bioactive (e.g., hemolytic and cytolytic) properties [3], and their action mechanisms are not always fully understood [4]. 

In Cnidaria, scyphozoan jellyfish (class Scyphozoa) are represented by nearly 220 species and can be found across all of the world’s oceans: although some occur in deep seas, most species live in shallow coastal habitats. Several scyphozoan jellyfish can reach conspicuous size and may seasonally undergo massive population outbreaks, resulting in large gelatinous biomasses being frequently detected in coastal waters, thus making these organisms ideal candidates for bioprospecting. In fact, several compounds with multiple biological properties have been isolated from the different body parts and components (e.g., tentacles, oral arms, umbrella, and secreted mucus) of several scyphozoan jellyfish [5,6,7]. For instance, peptides from the tentacles of the edible jellyfish *Rhopilema esculentum* were shown to have both ACE inhibitory and antioxidant abilities [8,9]. Extracts from whole specimens of the Mediterranean jellyfish *Cotylorhiza tuberculata*, *Rhizostoma pulmo,* and *Aurelia* *coerulea* show notable antioxidant activities [10]. Peptides derived from the pepsin-hydrolysed proteins (including collagen) of *R. pulmo* show remarkable antioxidant activity that is inversely proportional to the peptide molecular weight (MW), with low MW peptides being effective against oxidative stress in human epidermal keratinocyte (HEKa) cell cultures [11]. The antioxidant properties of *R. esculentum* extracts are also responsible of an in vivo photoprotection ability [12,13].

Bioactive compounds are present not only within jellyfish organs, tissues, and cells but also in secreted mucus. Indeed, jellyfish release considerable amount of mucus, an aqueous secretion, entangling a network of proteins and polysaccharides into a weak watery gel. As a dynamic external layer, mucus plays a variety of specialized functions across the animal kingdom [14], facilitating, for instance, locomotion, feeding, external case building, egg brooding, and osmolarity control as well as defence against predators, parasites, and microbial pathogens [15,16,17]. The epidermis of cnidarians is rich in gland-secretory cells, which are responsible of mucus production [18]. In *Aurelia* spp., mucus cells are abundant within the gastric pouches and the inner margin of the gonads, where mucus creates a defensive barrier into which antimicrobial compounds are released [17]. Antimicrobial peptides (AMPs) are indeed evolutionarily ancient weapons of the innate immunity of invertebrates, the first line of defence against a variety of pathogens, including protozoa, fungi, bacteria, and viruses [19]. Marine organisms live in a microbe-rich environment; therefore, a comparable, yet unexplored array of AMPs must have evolved [20,21].

Among the best characterized antimicrobial enzymes, lysozyme is a lytic agent that is able to break the peptidoglycan of bacterial cell walls by hydrolysing the beta-1,4-glycosidic bond between N-acetylmuramic acid (NAM) and N-acetylglucosamine (NAG) and by damaging the integrity of bacterial cells [22,23]. Lysozyme is abundant in a number of secretions, including mucus. In cnidarians, lysozyme-like activity was found in the anthozoan *Actinia equina* [17] and in the scyphozoan *R. pulmo*, presumably as defence against the surrounding pathogens [24,25,26]. 

In this framework, we explored the antimicrobial and antioxidant potential of extracts from the moon jellyfish *Aurelia coerulea* von Lendenfeld, 1884, one of the most common jellyfish in coastal areas worldwide. It also occurs in the Mediterranean Sea, where it is particularly encountered in coastal lagoons, sheltered inlets, and artificial habitats, such as marinas [27,28]. The life history traits of several scyphozoans, cubozoans, and hydrozoans are characterized by complex life cycles that usually alternate between three generations: the larva, the post-larval polypoid stage, and the adult medusa stage. These features confer high asexual reproductive potentials to several species, leading to the formation of densely aggregated populations in short periods [29]. Indeed, jellyfish outbreaks represent an emerging and recurrent problem in worldwide coastal areas, and these outbreaks are favoured by the rise of sea surface temperatures [30,31,32,33]. Due to the increased frequency and abundance of their population outbreaks, the scyphomedusae *A. coerulea*, *C. tuberculate,* and *R. pulmo* are increasingly considered valuable candidates for the exploitation of their biomasses and for the bioprospecting of compounds with pharmaceutical, nutritional, and nutraceutical value [10,24,25,26,34,35]. Further, jellyfish fishery can represent a sustainable activity to support biotechnology and recycling options [36]. Here, we report novel experimental data corroborating the antioxidant and antibacterial properties of extracts from the moon jellyfish *A. coerulea*, creating encouraging opportunities for the potential exploitation of non-indigenous species.

## 2. Results

### 2.1. Jellyfish Sample Characterization 

**Figure 1:**
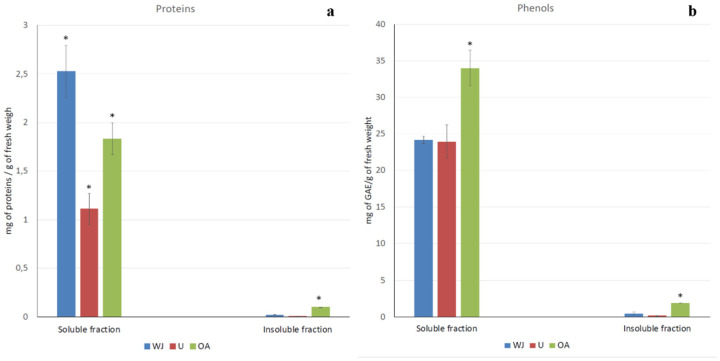
Total protein (**a**) and phenol (**b**) contents in the soluble and insoluble fractions of the jellyfish samples (WJ, whole jellyfish; U, umbrella; OA, oral arms). Protein content is expressed as mg of proteins per gram of fresh sample. Total phenolic content is expressed as mg of gallic acid equivalents (GAE) per gram of fresh weight. Data are means of three independent experiments performed in triplicate; bars represent ± standard deviation (SD). (* *p* < 0.05).

The protein content in the *A*. *coerulea* samples were evaluated, and the results are shown in Figure 1a. Both the fractions containing the soluble and insoluble compounds of the whole jellyfish (WJ) and the separated body parts, umbrella (U), and oral arms (OA) were considered. Most of the proteinaceous compounds were found in the soluble fraction, where the protein was 2.53 mg/g of fresh weight (FW), while only 0.02 mg/g of FW was insoluble in aqueous solution. Most of the proteins, both soluble and insoluble, were in the oral arms compared to being in the umbrella. Indeed, the soluble proteins were 1.83 mg/g of FW in the oral arms and 1.11 mg/g of FW in the umbrellas, and 0.10 mg/g and 0.01 mg/g were the FW of the proteins in insoluble fractions in the oral arms and umbrellas, respectively (Figure 1a). 

The phenolic compound content in the tissues of the *A. coerulea* samples was also evaluated and are showed in Figure 1b. The total phenol content was evaluated in the soluble and insoluble fractions of the whole jellyfish (WJ), umbrellas (U) and oral arms (OA). As for the protein content, phenolic compounds were more concentrated in the soluble fractions of all of the samples and were significantly higher in the oral arms (34.00 mg of gallic acid equivalents (GAE)/g of per 100 g of fresh weight (FW)) than in the umbrella (23.96 mg of GAE/g of FW). No significant differences were found between the phenolic content of the umbrella and the whole jellyfish (24.18 mg of GAE/g of FW). The insoluble fractions again were very poured in terms of phenolic compounds with, the most concentrated sample being in the oral arm (1.90 mg of GAE/g of FW) compared to both the umbrella (0.15 mg of GAE/g of FW) and the whole jellyfish (0.15 mg of GAE/g of FW).

### 2.2. Antioxidant Activity

**Figure 2:**
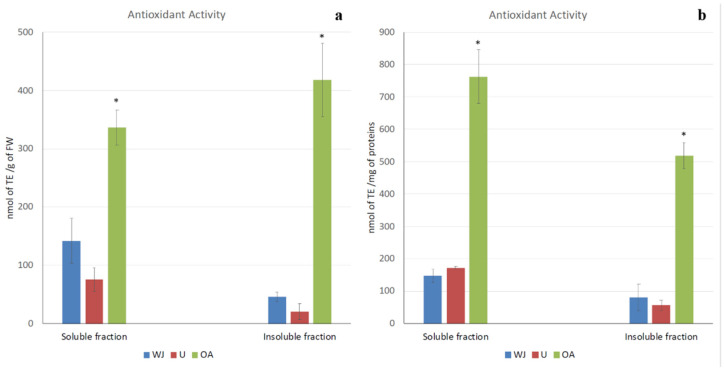
Total antioxidant activity in *Aurelia coerulea* jellyfish samples. Soluble and insoluble fractions of the whole jellyfish (WJ), umbrellas (U), and oral arms (OA) were considered. Antioxidant activity is expressed as nmol of Trolox eq. (TE) per g of fresh weight (**a**) or as nmol of Trolox eq. (TE) per mg of proteins (**b**). Data are the mean of three independent experiments performed in triplicate; bars represent mean ± standard deviation. (* *p* < 0.05).

Remarkable antioxidant activity, which could be measured as radical scavenging activity, was detected in all of the jellyfish samples, as shown in Figure 2a,b. Antioxidant activity referred to both the fresh weight of the jellyfish and the protein content in order to avoid artifacts related to sample concentration. The results were consistent; indeed, most of the antioxidant capacity was detected in both the soluble and insoluble fractions of the oral arm samples (762.32 nmol of TE/mg of proteins and 518.82 nmol of TE/mg of proteins, respectively). Very low antioxidant activity was measured in the umbrella and in the whole jellyfish in both the soluble (172.27 nmol of TE/mg of proteins and 147.35 nmol of TE/mg of proteins, respectively) and insoluble fraction (55.64 nmol of TE/mg of proteins and 80.29 nmol of TE/mg of proteins, respectively). No significant differences were detected between the antioxidant activity of the umbrella and the whole jellyfish.

#### SDS-PAGE Separation of Jellyfish Proteins

**Figure 3:**
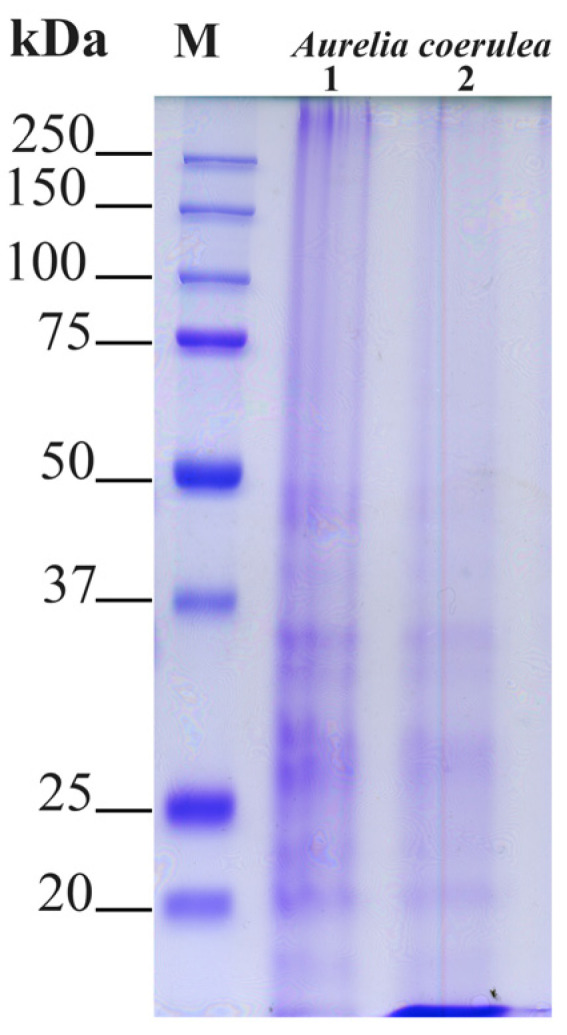
Polypeptide patterns of *Aurelia coerulea* umbrella (**1**) and oral arm (**2**) proteins separated by 12% reducing sodium dodecyl sulfate-polyacrylamide gel electrophoresis (SDS-PAGE). The molecular weight size marker (range of 250–15 kDa) was run in parallel with samples for molecular weight estimation. Each lane contained 20 μg of proteins and bands were visualized by staining gels with Coomassie Brilliant Blue R-250 dye.

Proteins of the different jellyfish samples were analyzed by SDS-PAGE electrophoretic separation. In Figure 3, each lane was loaded with 20 μg of protein, and the electrophoretic separation pattern was largely overlapping, apart from a slightly very low molecular weight component that did not separate in the 12% gel. Patterns of soluble polypeptides with a size higher than 20 kDa were compared in order to verify if differences in the electrophoretic pattern among the umbrella (1) and oral arms (2) are visible. As expected, no major differences were evidenced by the electrophoretic analysis, which revealed major protein bands ranging from 20 to 50 kDa and barely visible bands at low molecular weights less than 20 kDa, which were mainly visible in the oral arm samples.

### 2.3. Lysozyme-Like Activity

**Figure 4:**
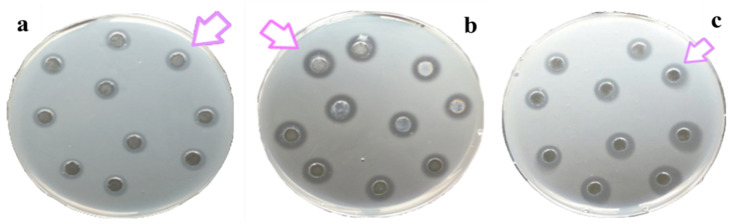
Standard assay on Petri dish inoculated with *Micrococcus luteus* cell walls to detect the lysozyme-like activity of *A. coerulea* mucus (**a**), oral arms (**b**), and umbrella (**c**). The arrows indicate the lysis diameter around each well.

All of the considered compartments, namely the mucus, oral arms, and umbrella of *A. coerulea,* showed natural lysozyme like activity (Figure 4). By a standard assay on Petri dishes, a lysis diameter of 3.1 ± 0.5 mm corresponding to 0.41 mg/mL of hen egg-white lysozyme was observed in the mucus compartment; a diameter of lysis of 5.6 ± 0.5 mm corresponding to 0.76 mg/mL of hen egg-white lysozyme was detected in the oral arms compartment; and a diameter of lysis of 4.0 ± 0.4 mm corresponding to 0.54 mg/mL of hen egg-white lysozyme was recorded for the umbrella compartment.

#### 2.3.1. Effect of pH on Lysozyme-Like Activity

The natural lysozyme-like activity varied significantly across the investigated compartments of *A. coerulea* and was strictly dependent on the different pH values (Table 1).

**Figure 5:**
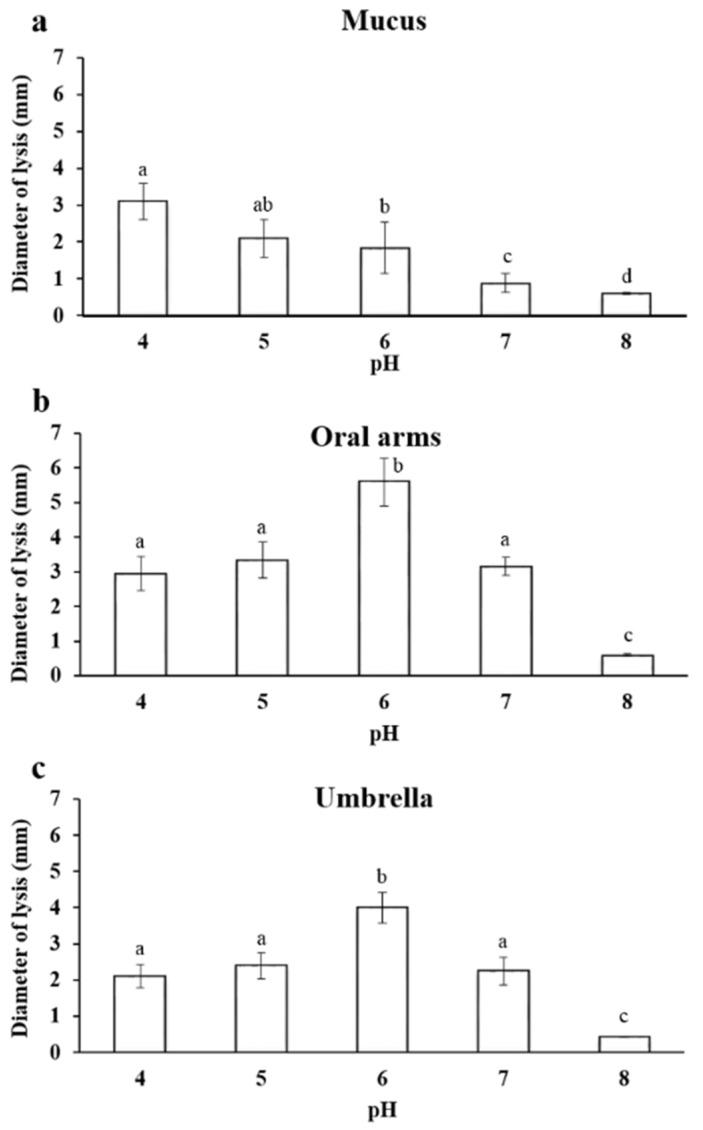
Effect of pH (4, 5, 6, 7, 8) on the lysozyme-like activity of *A. coerulea* mucus (**a**), oral arms (**b**), and umbrella (**c**). Data are reported as mean value ± standard deviation. Sharing letters indicate absence of significant differences.

The lysozyme-like activity was strongly affected by the pH of the *A. coerulea* mucus samples and a decrease of the lysis diameter was observed when the pH increased (Figure 5a). In particular, the maximum activity was observed at pH 4.0, and instead, the minimum lysis diameter was 0.6 ± 0.03 mm at pH 8.0.

Additionally, in the homogenate of the oral arms, the lysozyme-like activity was strongly influenced by the pH of the sample and by the reaction medium. In particular, the minimum lysis diameter was 0.60 ± 0.03 mm at pH 8.0. The highest activity was instead observed at pH 6.0 (Figure 5b).

The lysozyme-like activity of the umbrella homogenate was strictly affected by the pH. In particular, the lowest lysis diameter was 0.6 ± 0.02 mm at pH 8.0. The highest activity was observed at pH 6.0 (Figure 5c).

#### 2.3.2. Effect of Ionic Strength on Lysozyme–Like Activity

All of the examined compartments of *A. coerulea* showed natural lysozyme-like activity that was strictly affected by the ionic strength (I) of the sample and of the reaction medium (Figure S1), reaching the maximum with a value of ionic strength equal to 0.175.

#### 2.3.3. Effect of Temperature on Lysozyme–Like Activity

**Figure 6:**
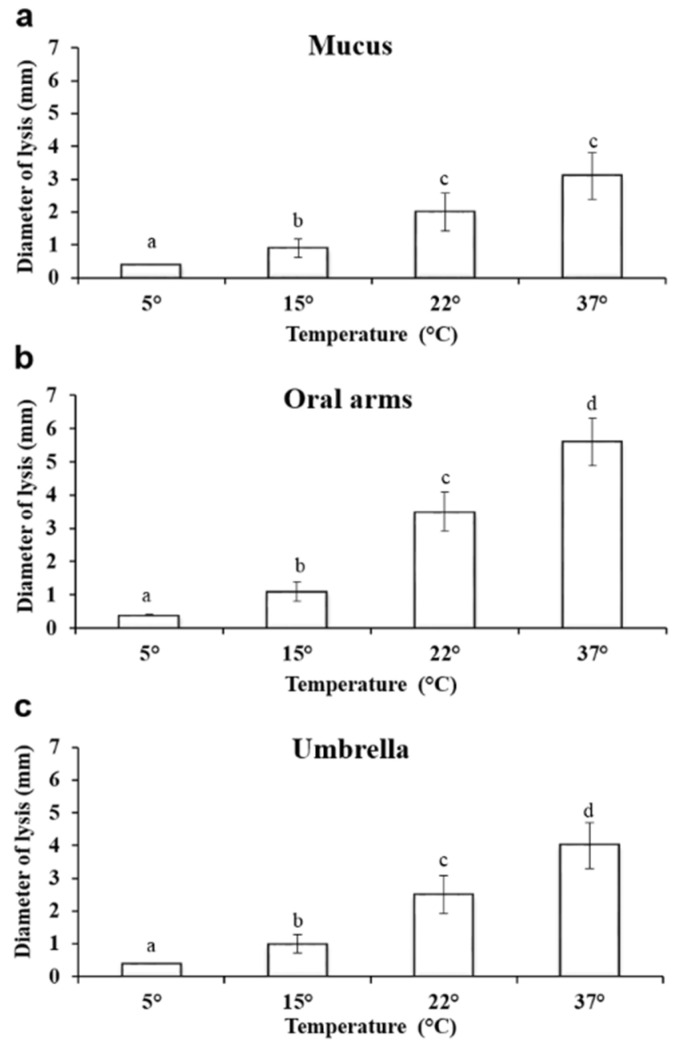
Effect of the incubation temperature on the lysozyme-like activity of *A. coerulea* mucus (**a**), oral arms (**b**), and umbrella (**c**). Data are reported as mean value ± standard deviation. Sharing letters indicate absence of significant differences.

The effect of temperature on the lysozyme-like activity was also evaluated. At 5 °C, the lysis diameter was 0.4 ± 0.002 mm in the mucus compartment, while at 15 °C, the lysis diameter was 0.9 ± 0.2 mm. When the test was conducted at 37 °C, the lysozyme-like activity of the oral arms was the highest measured (Figure 6).

The results of the PERMANOVA tests revealed that the natural lysozyme-like activity varied significantly across the investigated compartments of *Aurelia* at different temperature conditions (Figure 6; Table 2).

At 5 °C, the lysis diameter was 0.4 ± 0.002 mm in the mucus compartment, while at 15 °C, the lysis diameter was 0.9 ± 0.2 mm. When the test was conducted at 37 °C, the lysozyme-like activity of the mucus was the highest measured, reaching 3.1 ± 0.7 mm (Figure 6a).

Additionally, in the oral arms homogenate, a significant increase in the lysis diameter was observed when the temperature increased (Figure 6). At 5 °C, the lysis diameter was 0.4 ± 0.1 mm. At 15 °C, the lysis diameter was 1.1 ± 0.2 mm, and at 22 °C, it was 3.5 ± 0.42 mm. The largest lysis diameter (5.6 ± 0.5 mm) was recorded at 37 °C (Figure 6b).

A similar trend was also observed in the umbrella homogenate, with a significant increase in the lysis diameter being recorded when the incubation temperature increased (Figure 6). At 5 °C, the lysis diameter was 0.4 ± 0.07 mm, and at 15 °C, it was 1.0 ± 0.2 mm and 2.5 ± 0.4 mm at 22 °C. The largest lysis diameters were recorded at 37 °C, reaching a value of 4.0 ± 0.5 mm (Figure 6c).

**Figure 7:**
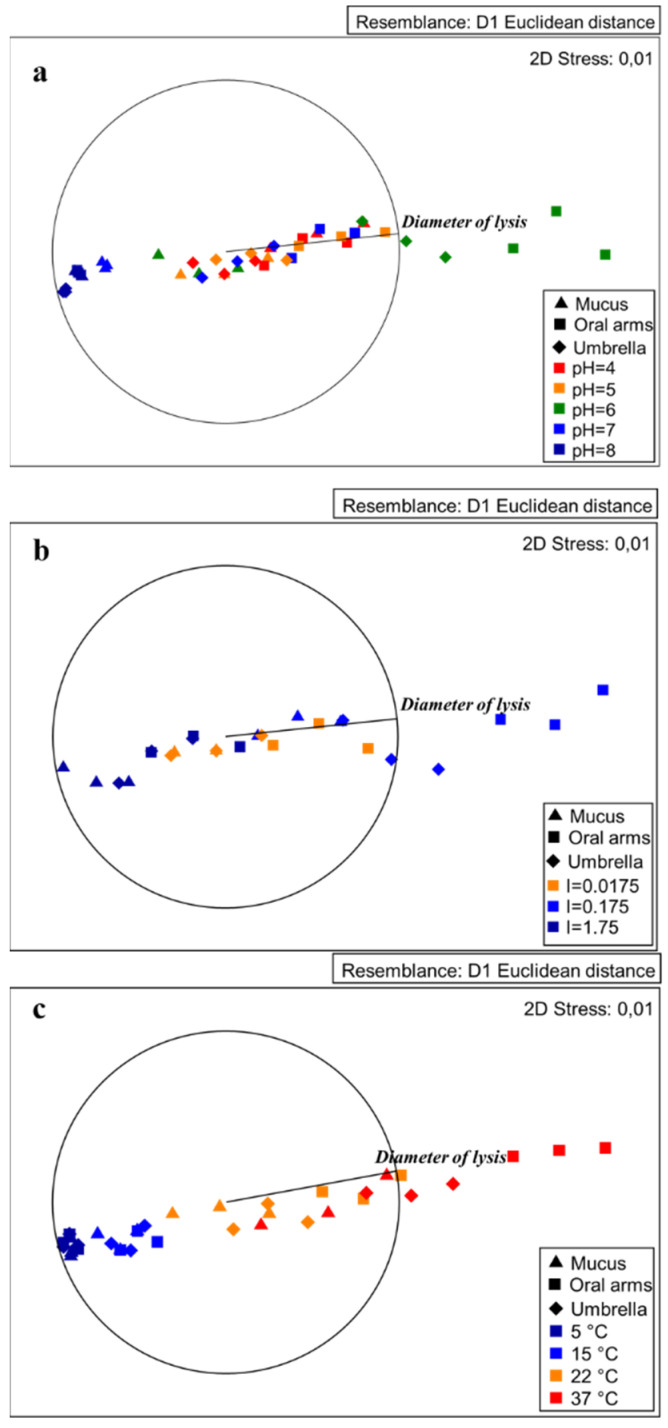
MDS plot of the lysozyme-like activity detected in the *A. coerulea* mucus, oral arms, and umbrella homogenates showing the effects of pH (**a**), ionic strength (**b**), and temperature (**c**) on the lysis diameter.

The MDS plots showed some clear segregations among the tested conditions. In particular, the conditions producing worthy lysozyme-like activity seemed to be at pH 6, with an ionic strength corresponding to 0.175 and a temperature of 37 °C for both the oral arms and the umbrella (Figure 7). In the following subsections, the pH, ionic strength, and temperature effects are described.

**Figure 8:**
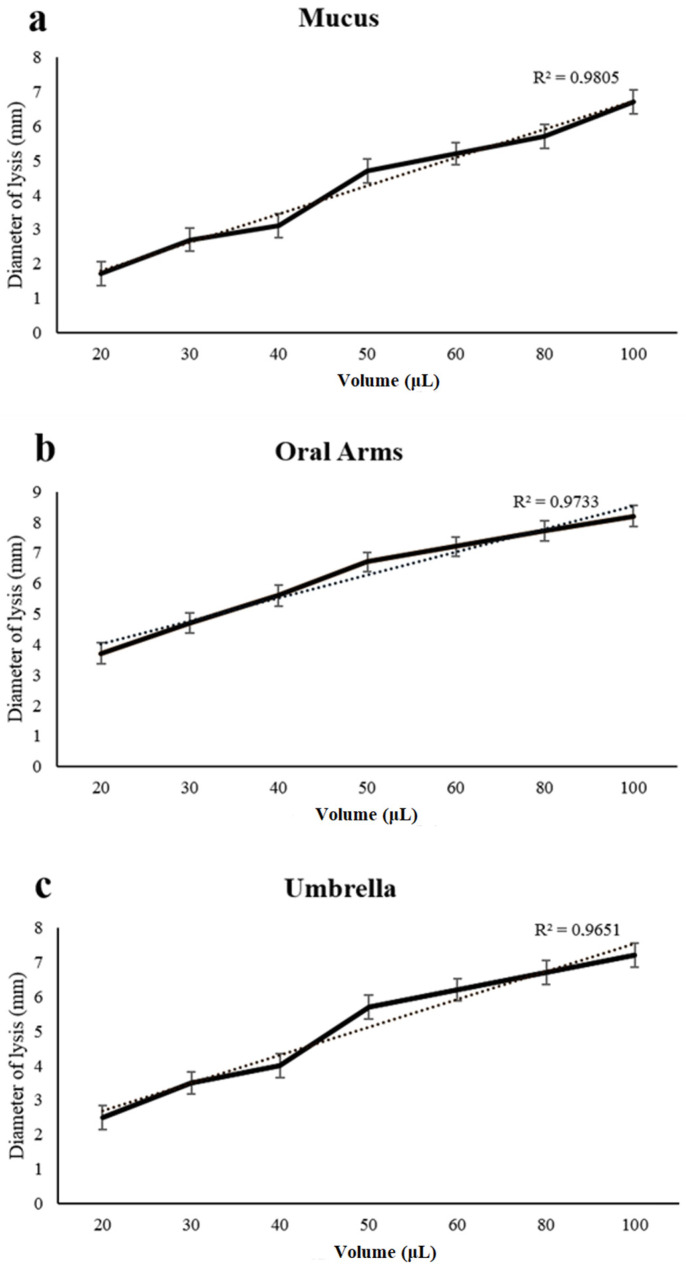
Dose–response curve of lysozyme-like activity recorded in the *A. coerulea* mucus (**a**), oral arms (**b**), and umbrella (**c**) homogenates. Wells in a Petri dish were filled with different volumes of sample (from 20 to 100 μL). Values are given as means ± standard deviation.

A dose–response curve was obtained when increasing the amounts of the mucus, oral arms, and umbrella, respectively, which were used to test the lysozyme-like activity (Figure 8). The diameter of the lysis was positively correlated with the employed volume of the mucus, oral arms, and umbrella samples (Figure 8).

## 3. Discussion

Linked to the human-driven spread of the Pacific oyster worldwide, the moon jellyfish *Aurelia coerulea* (first described from the Pacific coasts of Australia and the Sea of Japan) is now regarded as a cosmopolitan jellyfish [27]. For more than 40 years, this species has been able to be found in most Western and Central Mediterranean coastal lagoons, with seasonal population outbreaks. Their outbreaks represent an emergent environmental issue in many coastal areas, with negative impacts on both ecosystem functioning and human activities. In particular, *Aurelia* spp. blooms occur in the Mediterranean Sea, from Spanish to the North Adriatic coasts yearly, providing considerable biomass from large populations of jellyfish, which has so far been rarely investigated from a Blue Biotechnology perspective [10]. However, the exploitation of jellyfish populations may be envisaged as a potential source for the isolation and sustainable production of natural compounds in several fields including, biotechnological and pharmaceutical applications, as demonstrated also by the results of the present work. Indeed, we corroborate here the biotechnological potential of *A. coerulea*, highlighting the occurrence of potent antioxidant and antibacterial (lysozyme-like) compounds that are associated with the jellyfish mucus, oral arms, and umbrella.

The evidence for bioactive compounds with antioxidant activity in *A. coerulea* is intriguing when considering that natural antioxidant compounds play an important role as health-protecting factors from free radicals and ROS (reactive oxygen species) effects. Compounds with antioxidant activity are predicted to have other bioactivities and health benefits that are directly or indirectly linked to antioxidant property, such as anti-inflammatory, anticancer, anti-aging, and protective action for cardiovascular diseases, diabetes, obesity, and neurodegenerative diseases; all of the applications have a dysregulation of the oxidative balance at their base [37]. Then, interest in natural antioxidants has increased because they are widely distributed and safer than synthetic antioxidants; other than terrestrial plants, the marine environment represents a source of natural bioactive molecules that are still poorly explored. Previous and ongoing research has demonstrated the presence of strong antioxidant activity in proteinaceous and non-proteinaceous extracts from different species, including non-native jellyfish in the Mediterranean Sea, such as Red Sea immigrant *Cassiopea andromeda* [10,11,38,39,40]. The antioxidant activity is stable and heat resistant when associated with peptides or to phenolic compounds [10,11]. The phenolic compounds that are widespread between plants and microorganisms are some of the most effective antioxidants known in nature [41,42,43] and have also been found also in jellyfish [10,11,38,40]. In *A. coerulea*, we demonstrated that conspicuous antioxidant activity was exerted by oral arms and umbrella tissues; however, antioxidant compounds seem more concentrated in the oral arms despite the low content of both proteins and phenolic compounds. From these data, low molecular weight components—mainly present in the oral arms—seem to have the highest antioxidant activity. However, the SDS-PAGE (and the resulting separation of polypeptides) is only the first step in the analysis of the proteinaceous components, which is not necessarily predictive of any biological activity. Eventually, an *in-depth* analysis will be required for the identification of the proteins/peptides or other molecules that are responsible for the observed biological activities. We hypothesize that the compounds evidenced from *A. coerulea* may have biological activities linked to defensive mechanisms: however, on the basis of our data, no clear relationship between the antioxidant and antibacterial activities can be established, although several peptides and phenolic compounds are known to exert both activities [44].

Many marine taxa, including cnidarians, are known to develop defence mechanisms and to synthesize an astonishing variety of bioactive compounds [45], including antioxidant and antibacterial compounds such as lysozyme [46] to prevent the settlement and growth of microbial agents [47,48].

The occurrence of lysozyme-like enzymes is known in diverse marine invertebrates [49,50,51,52], including in cnidarian tissues [51,52], isolated oocytes, [24] and mucus [17]. However, all of the previous studies dealt with a single compartment or with the whole animal. The present paper is the first report on the lysozyme-like activity (testing the ability to lyse in vitro of the cell wall of the bacterium *Micrococcus luteus*) comparatively detected from three different compartments of the same jellyfish species, namely *Aurelia coerulea* umbrella, oral arms, and mucus.

Lysozyme represents the best characterized enzyme that is involved in the self-defence from bacteria and has been suggested as a rudimentary molecular defence, especially in organisms without adaptive immunity [53,54]. This enzyme can perform its function as an antibacterial agent through both direct and indirect lytic action on bacteria (e.g., on the stimulation of phagocytosis) [54], although different roles in protective reactions are likely. Eventually, further research is required and will be undertaken soon in order to evaluate whether these compartments may prove to be effective on other microorganisms other than *Micrococcus luteus,* such as *Escherichia coli* and *Pseudomonas aeruginosa*.

Here, the protein pattern of the A. *coerulea* detected by electrophoretic analysis indicates the occurrence of proteins ranging from 20 to 50 kDa. This is in agreement with the general multi-protein nature of other marine invertebrate body compartments and with the previous SDS-analysis of *Aurelia* jellyfish in Leone et al. [10], indicating that a pool of proteins and peptides can be part of the jellyfish defence system. In *Chrysaora quinquecirrha,* toxic proteins have been categorized as medium-sized cytolytic actinoporins (∼20 kDa), cardiostimulatory proteins (∼28 kDa), and cytolysin with or without phospholipase in both crude and fractionated proteins (∼40 kDa) [51]. In *Chrysaora achlyos,* major protein component venom was found at 55 kDa [52]. The lysozyme activity recorded in *A. coerulea* in the present study could be ascribed to one the major protein bands evidenced by the electrophoretic analysis. Accordingly, research focused on the structure and function of lysozymes from several bivalve molluscs showed a molecular weight ranging from 11 to 22 kDa [55,56,57]. In the present study, we observed different features of the lysozyme activities in the examined *A. coerulea* compartments. In particular, in the *A*. *coerulea* umbrella and oral arms, the lysozyme had a maximum of activity when the pH of both the reaction medium and the sample was 6 and when the ionic strength was 0.175, as previously reported for the lysozyme present in the mucus and in the extract of the nematocysts of *A. equina* and for other lysozymes [17]. The maximum lysis diameter observed in *A. equina* nematocysts (L. Stabili, unpublished data) is equal to 4.75 ± 0.7 mm, which is very close to the lysis diameter produced by the lysozyme-like activity evidenced in the homogenate of the *A*. *coerulea* oral arms and umbrella. Moreover, in the present study, the obtained results prove that the homogenate of oral arms had lysozyme-like activity that was higher than that of umbrella and mucus. This could represent a further support within jellyfish tentacles as defence mechanism that is constituted by millions of nematocysts that are ready to trigger when physical contact occurs [58,59,60]. However, thanks to the conspicuous biomasses of the *A. coerulea* umbrella compared to the other body compartments, the potential exploitation of the entire jellyfish could be encouraged.

Regarding mucus in marine invertebrates, it has a wide range of attributes and functions, including defence against an array of environmental stressors such as predators, parasites, and pathogens [15,61]. As suggested by Calow [62], mucus could be affected by microbial attacks. Some invertebrates seem to adopt some advantageous strategies to inhibit bacterial attack, combining their mucus with antibiotic molecules or preventing bacterial growth thanks to mucus that is poor in proteins [63]. The antibacterial activity present in the mucus of *A. coerulea* could be due to the self defence of the jellyfish and the secretion of lysozyme in this matrix or could be due to the release of lysozyme by bacteria hosted in the mucus. In this last case, this could explain the different characteristics of the lysozyme activity recorded in the umbrella and oral arms (with a maximum of activity at pH 6) and the activity observed in the mucus that showed a maximum of activity when the pH of the reaction medium and sample was 4 and when the ionic strength was 0.175. Indeed, feeding may not be the only activity involved in the association of microorganisms with the mucus since, as already observed in sponges and bryozoans, the bacterial symbionts present in the mucus may produce chemicals that protect their hosts against potential pathogenic bacteria [64,65,66]. In the case of coral-associated bacteria, for example, it has also been hypothesized that they play a role in the host resistance to disease by competing for nutrients and/or space with other hazardous microorganisms to the host and by producing antibiotics [67,68,69]. However, independently from the origin, the presence of lysozyme in the mucus of *A. coerulea*. indicates the role of this compartment in defending the jellyfish from bacterial attack, serving as a medium into which the antibacterial substances are exuded. The defensive role of mucus has already been evidenced in several marine invertebrates, including polychaetes, such as *Sabella spallanzanii* and *Myxicola infundibulum* [70,71], and in several corals [72]. This role is fundamental, taking into account that *A. coerulea* lives along the coastal lagoons and harbours where bacteria, which often include pathogens that are harmful to man and marine organisms, are copious. Although bacteria may colonize the jellyfish, our results suggest the presence of a powerful immune system that includes lysozyme-like activity.

In recent years, lysozyme has attracted the interest of researchers due to its potential applications in the pharmaceutical and aquaculture fields [73]. Currently, the search and use of antimicrobials that are able to treat pathologies and specific diseases is necessary to guarantee the health of farmed animals and to encourage safer and more sustainable aquaculture [74]. Therefore, to improve the therapeutic effectiveness in aquaculture, new formulations prepared by the association of lysozyme to vaccines obtained from *Vibrio anguillarum* and *Edwardsiella piscicida* have been proposed [75]. In this framework, *A. coerulea* could represent a new natural source of lysozyme-like compounds.

Currently, lysozyme is also used for pharmaceutical preparations [76,77,78]. Lysozyme can be used against a wide array of infections in humans, such as gastrointestinal, paediatric, ophthalmic, and oral infections. Furthermore, since does not have any toxic effects on humans, lysozyme is a good candidate for the use of epidermal and cosmetic formulations. The results of the present study showing the presence of lysozyme-like activity in all of the examined extracts of A. *coerulea* encourage the potential use of the jellyfish for lysozyme-based preparations that are also in the pharmaceutical field.

Recently, a notable discovery of antimicrobial peptides and new thermo-stable proteases was achieved from the sea anemones *Actinia equina* and *Anemonia sulcata*, which had important application perspectives for the discovery of new biocleaning or antifouling agents [79]. We believe *A. coerulea* may represent an additional source of compounds for the development of natural antifouling molecules. This is particularly important considering that, up to now, highly toxic biocides are used [80,81,82,83].

In conclusion, this study corroborates previous findings encouraging the possible exploitation of *A. coerulea* (and other jellyfish species) for biotechnological applications due to the evidence of antioxidant and antibacterial activities. A metabolomic approach based on advanced technologies, such as NMR resonance spectroscopy, will rapidly allow the multicomponent detection of complex mixture obtained from *A. coerulea* different body compartments. In this framework, further studies will also be required to isolate molecules of the utmost biotechnological interest through the combination of different analytical techniques, such as the HPLC, GC–MS, and LC–MS methods.

## 4. Materials and Methods

### 4.1. Sample Collection and Preparation

**Figure 9:**
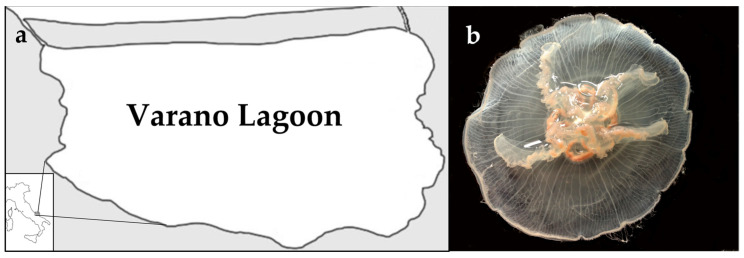
Varano lagoon (**a**). A specimen of *Aurelia coerulea* (**b**) collected in the study area.

Varano Lake (41° 52′45.01″ N, 15°44′46.00″ E) is located on the southern Adriatic Sea on the north side of the Gargano promontory (Apulia region, Italy, Figure 9). The lagoon area covers approximatively 60.5 km^2^ and reaches a maximum depth in the central zone of 5 m. Here, a resident population of *Aurelia coerulea* was recorded throughout the year, with high densities of jellyfish being present in spring and summer months, when the population abundance was estimated to be 4.5 ind/m^2^ [28].

Fifty specimens of *A. coerulea* adult medusae were sampled from a boat using a 1 cm mesh net. Immediately after sampling, jellyfish specimens were transported to the laboratory for the processing. In the laboratory, the animals were washed using filter-sterilized seawater (0.2 µm, Millipore, Bedford, MA, USA to remove the mucus layer produced during transport and were then placed in sterilized containers. The secreted mucus (M) produced in controlled conditions was collected using a sterile Pasteur pipette and was stored at −80 °C for the lysozyme-like activity assays. Subsequently, during mucus collection, some jellyfish were dissected by separating the oral arms from the umbrella. Whole jellyfish (W), the oral arms (OA), and umbrellas (U) were either frozen in liquid nitrogen as subsequently described or were directly homogenized for 90 s at 16,000 rpm in a sterile Waring blender at 4 °C and employed for further analyses. Some specimens were frozen in liquid nitrogen and stored at −80 °C.

### 4.2. Protein, Phenolic and Antioxidant Analyses

Whole jellyfish (W) and the separated oral arm (OA) and umbrella (U) specimens were homogenized in a sterile Waring blender that had previously been refrigerated. The entire procedure was performed at 0–4 °C by keeping the samples on ice. Homogenate samples were poured in 50 mL sterile Falcons (Corning, NY, USA) and were centrifuged at 9000× *g* for 30 min at 4 °C. The supernatants, which contained soluble compounds, including soluble proteins, were separated from the insoluble material remaining in the pellet, which included insoluble proteins, such as fibrillar collagen, and other insoluble components. Both fractions were analysed for the protein content and phenolic compounds as well as for the antioxidant activity.

#### 4.2.1. Protein Content

Total protein content was estimated in each sample through the modified Bradford assay (Bradford, 1975) using bovine serum albumin (BSA) as a standard.

#### 4.2.2. Phenol Content

The total phenol content was evaluated in soluble and insoluble fractions of samples of whole (W) jellyfish as well as in separated umbrella (U) and oral arms (OA) samples by a modified Folin–Ciocalteu colorimetric method as described in Leone et al. [10]. Briefly, samples (100 μL) were mixed with 500 μL of Folin–Ciocalteu’s phenol reagent and 500 μL of 7.5% sodium carbonate (Na_2_CO_3_). After 2 h of incubation at room temperature in the dark, the absorbance was spectrophotometrically measured at 760 nm. Gallic acid, ranging from 25 to 200 μg/mL, was used as a standard. The results were expressed as gallic acid equivalents (GAE) per gram of dry extract.

#### 4.2.3. Antioxidant Activity

The total antioxidant activity was determined spectrophotometrically in each sample by using the Trolox Equivalent Antioxidant Capacity (TEAC) method, as described by Longo et al. [84]. Briefly, 10 μL of the jellyfish samples were assayed in 1 mL of the reaction mixture, and the depletion of the radical cation ABTS•+ was measured following the decrease of absorbance at 734 nm. Comparable solutions of PBS, seawater, and bi-distilled water were used as controls. A calibration curve was prepared with different concentrations of Trolox (2.5–20 μM). The antioxidant capacity of the samples was calculated as the absorbance decrease at 734 nm at 6 min as the fixed time, and the results were expressed as the nmol of Trolox equivalents (TE) per gram of sample or per mg of contained proteins.

#### 4.2.4. SDS-PAGE Analysis

Proteins from the lyophilized samples (100 mg) of total jellyfish, umbrella, and oral arms were analysed by SDS-PAGE as in Leone et al. [10], with slight modifications. Lyophilized jellyfish samples were quickly washed twice with 16 volumes (*w*/*v*) of cold PBS by stirring and were then centrifuged at 9000× *g* for 30 min at 4 °C in order to eliminate any remaining salts. Proteins were solubilized in Laemli buffer 6× and were analysed by SDS-PAGE.

Briefly, polypeptides were separated by electrophoresis through 12% polyacrylamide gels containing SDS (SDS-PAGE), as described in Leone et al. [10]. Twenty-five micrograms of jellyfish proteins from total and umbrella’s jellyfish and twenty micrograms from the oral-arm proteins were separated, and the Precision Plus Protein Dual Color Standard (Bio-Rad, Hertfordshire, UK) was used as a molecular weight marker. Polypeptides on gels were detected by Coomassie Brilliant Blue staining (0.25% Coomassie Brilliant Blue R-250 in 10% acetic acid and 50% methanol) for 20 min followed by destaining with 10% acetic acid and 30% methanol overnight. Molecular masses of the proteins were estimated by comparing the migration of the proteins of interest to the standards of known sizes.

### 4.3. Lysozyme-Like Activity

The presence of lysozyme activity can be detected by several methods. In the present paper, the occurrence of lysozyme activity was detected using the standard assay on Petri dishes, as already recently performed in other studies on Cnidarians in order to compare the data obtained in different species [17,28,85]. Dishes were prepared according to the following protocol: 700 µL of 5 mg/mL of peptidoglycan from *Micrococcus luteus* (Sigma, Saint Louis, MO, USA) were suspended in 7 mL of 0.05 M PB-agarose (1.2%, pH 5.0) and then spread on Petri dishes. Wells with 6.3 mm diameters were sunk in the agarose gel, and each well was filled with 30 µL of sample (mucus, oral arms, and umbrella, respectively). The diameter of the cleared zone of at least five replicates was recorded after overnight incubation at 37 °C. Diameters of lysis were compared with those of reference obtained with known amounts of standard hen egg-white lysozyme (Merck, Darmstadt, Germany). The effects of pH, ionic strength (I), and temperature were evaluated for each sample. The pH effect was established by dialyzing (7000-MW cut-off) the samples in PB 0.05 M, ionic strength, I = 0.175, which was adjusted at pH 4, 5, 6, 7, 8, and by dissolving agarose in PB at the same I and pH values. The ionic strength effect was tested in PB 0.05 M and was adjusted at I = 0.0175, 0.175, 1.75. Agarose was dissolved in PB at the same I values. The temperature effect was assessed by performing the Petri dish assays (in PB at I = 0.175 and pH 6.0 for OA and U and pH 4 for M) and by incubating the plates at 5, 15, 22, and 37 °C. The dose–response curve of lysozyme-like activity was produced using Petri dish assays (in PB, at I = 0.175 and pH 6.0 for OA and U and pH 4 for M) with different amounts of sample (20, 30, 40, 50, 60, or 100 µL of sample in each well in triplicate).

### 4.4. Statistical Analysis

To evidence significant differences in the amount of protein contents, phenol contents, and antioxidant activity, a T test was used. Randomization of the T test was chosen to cope with the possible problem of non-normality and the heteroscedasticity of the data. The comparisons were made both in the whole jellyfish tissue and in the separated body parts (umbrella and oral arms). Statistical analysis was conducted with the software R [86], using the package “boot” [87,88].

To test the effects of temperature, pH, and ionic strength on antibacterial activity of several body compartments, permutational analyses of variance (PERMANOVA) were performed based on Euclidean distances on untransformed data, using 9999 random permutations of the appropriate units [89,90]. Three different designs were adopted with two factors: i) compartment (Co, as fixed factor with three levels) and ii) temperature (Te, as fixed factor with four levels); or pH (pH, as fixed factor with five levels) or ionic strength (IS, as fixed factor with three levels) separately. Post hoc pairwise tests were performed when significant differences were found (*p* < 0.05) to verify the consistency of the differences among several different levels that were investigated. When the restricted number of unique permutations in the pairwise tests occurred, p values were obtained from Monte Carlo samplings. Differences were illustrated through multidimensional scaling (MDS) plots. The analyses were performed using the software PRIMER v. 6 [91].

## 5. Conclusions

Further investigations will be required in order to isolate the potential molecules of interest that are responsible for the antibacterial and antioxidant activities of the investigated moon jellyfish. Recent studies on the composition profile of proteins and metabolites in the homogenate of *A. coerulea* provided the first insights into the discovery of new compounds [10]. Our results highlighted that valuable bioactivity is associated with the compounds associated with the mucus, oral arms, and umbrella of the moon jellyfish *A. coerulea.* Usually considered a critical issue in coastal areas, this alien jellyfish may be differently valued under a Blue Growth perspective, as it is a potential novel source of antioxidant and lysozyme-like compounds with multiple potential applications in the biotechnological and drug discovery sectors.

## Figures and Tables

**Table 1:** Results of PERMANOVA testing for differences in lysozyme-like activity among compartments under tested pH conditions.

Source	df	MS	Pseudo-F	P(perm)
Co	2	7.79	60.55	
pH	4	12.78	99.32	
Co x pH	8	2.24	17.45	***
Res	30	0.13		
Total	44			

Co—compartment; pH—pH; Res—residual; Tot—total; df—degrees of freedom; MS—mean squares; Pseudo-F—F critic; P(perm)—permutational level of probability; *** *p* < 0.001.

**Table 2:** Results of PERMANOVA testing for differences in lysozyme-like activity among compartments under tested temperature conditions.

Source	df	MS	Pseudo-F	P(perm)
Co	2	3.40	24.91	
T	3	26.91	197.33	
Co x T	6	1.06	7.81	***
Res	24	0.14		
Total	35			

Co—compartment; T—temperature; Res—residual; Tot—total; df—degrees of freedom; MS—mean squares; Pseudo-F—F critic; P(perm)—permutational level of probability; *** *p* < 0.001.

## Data Availability

Data available on request from the authors.

## Supplementary Materials

The following are available online at https://www.mdpi.com/article/10.3390/md19110619/s1, Figure S1. Effect of the ionic strength on the lysozyme-like activity of *A. coerulea* mucus (a), oral arms (b), and umbrella (c). Data are reported as mean value ± standard deviation. Sharing letters indicates absence of significant differences.
