# Supplementary material for: Jellyfish Bioprospecting in the Mediterranean Sea: Antioxidant and Lysozyme-Like Activities from Aurelia coerulea (Cnidaria, Scyphozoa) Extracts
Source: Mar Drugs. 2021 Oct 31;19(11):619. doi: 10.3390/md19110619 (PMC8625557; doi:10.3390/md19110619)
Supplement: Supplementary file 1 [file marinedrugs-19-00619-s001.zip › marinedrugs-1413493-supplementary.pdf]

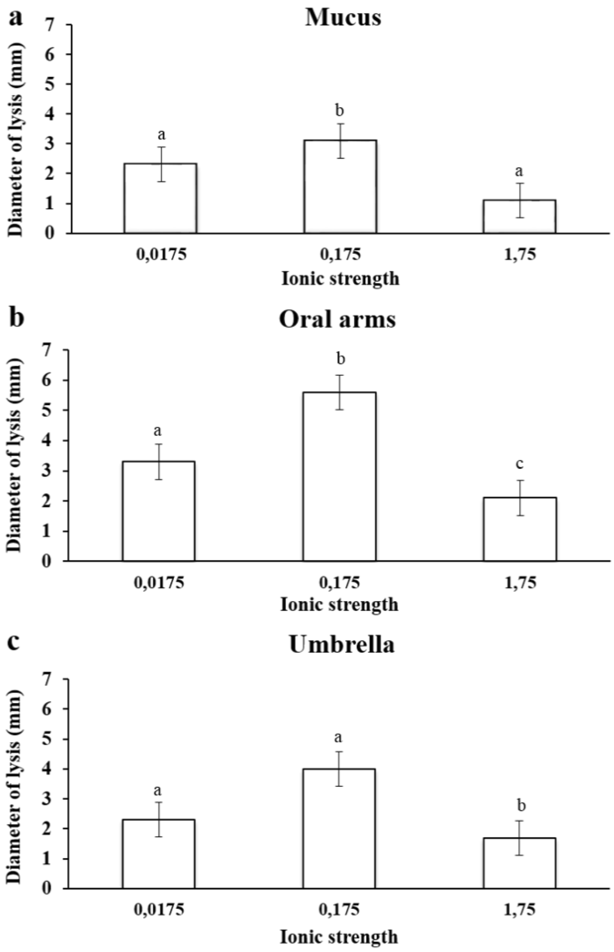

Figure S1. Effect of the ionic strength on the lysozyme-like activity of *A. coerulea* mucus (a), oral arms (b) and umbrella (c). Data are reported as mean value ± standard deviation. Sharing letters indicate absence of significant differences.
